# Viscosity Modeling for Blood and Blood Analog Fluids in Narrow Gap and High Reynolds Numbers Flows

**DOI:** 10.3390/mi15060793

**Published:** 2024-06-16

**Authors:** Finn Knüppel, Sasha Malchow, Ang Sun, Jeanette Hussong, Alexander Hartmann, Frank-Hendrik Wurm, Benjamin Torner

**Affiliations:** 1 Institute of Turbomachinery, Faculty for Mechanical Engineering and Ship Design, University of Rostock, 18059 Rostock, Germany; finn.knueppel@uni-rostock.de (F.K.); sasha.malchow@uni-rostock.de (S.M.); hendrik.wurm@uni-rostock.de (F.-H.W.); 2 Institute for Fluid Mechanics and Aerodynamics, Technical University of Darmstadt, 64287 Darmstadt, Germany; sun@sla.tu-darmstadt.de (A.S.); hussong@sla.tu-darmstadt.de (J.H.); 3 Institute of Clinical Chemistry and Laboratory Medicine, Rostock University Medical Center, 18057 Rostock, Germany; alexander.hartmann2@med.uni-rostock.de

**Keywords:** cell-free layer, Fåhræus–Lindqvist effect, blood, particle-laden blood analog fluid, viscosity modeling, CFD simulations

## Abstract

For the optimization of ventricular assist devices (VADs), flow simulations are crucial. Typically, these simulations assume single-phase flow to represent blood flow. However, blood consists of plasma and blood cells, making it a multiphase flow. Cell migration in such flows leads to a heterogeneous cell distribution, significantly impacting flow dynamics, especially in narrow gaps of less than 300 μm found in VADs. In these areas, cells migrate away from the walls, forming a cell-free layer, a phenomenon not usually considered in current VAD simulations. This paper addresses this gap by introducing a viscosity model that accounts for cell migration in microchannels under VAD-relevant conditions. The model is based on local particle distributions measured in a microchannels with a blood analog fluid. We developed a local viscosity distribution for flows with particles/cells and a cell-free layer, applicable to both blood and analog fluids, with particle volume fractions of up to 5%, gap heights of 150 μm, and Reynolds numbers around 100. The model was validated by comparing simulation results with experimental data of blood and blood analog fluid flow on wall shear stresses and pressure losses, showing strong agreement. This model improves the accuracy of simulations by considering local viscosity changes rather than assuming a single-phase fluid. Future developments will extend the model to physiological volume fractions up to 40%.

## 1. Introduction

Heart failure affects around 15 million people in Europe [1]. For advanced heart failure, heart transplantation remains the gold standard. However, many patients must endure prolonged waiting periods until a suitable donor heart becomes available, highlighting the imbalance between the supply and demand for donor hearts. This disparity is underscored by the Eurotransplant report, which reveals that only 629 heart transplants were performed, while 1019 patients remained on the waiting list [2].

**Figure 1:**
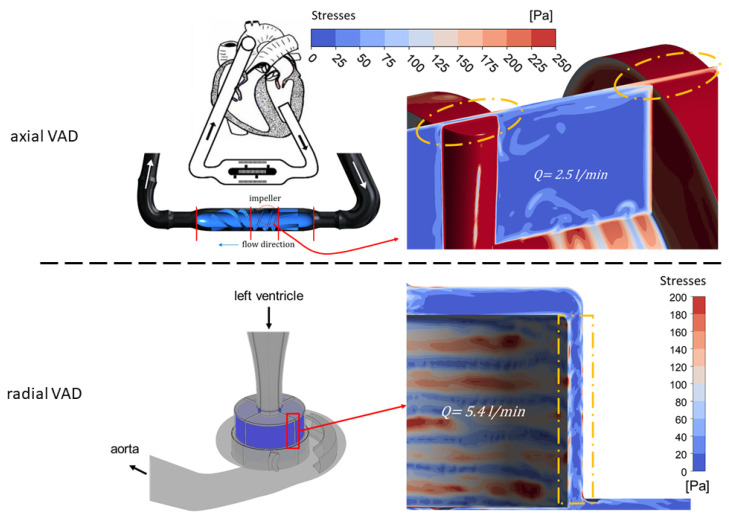
(**Top**): Axial VAD and stress field in the gaps between the impeller and outer housing. (**Bottom**): Radial VAD and stress field in the leakage flow in the side chambers between rotating impeller and stationary housing.

Fortunately, a technical solution for treatment is available in the form of ventricular assist devices (VADs). A VAD supports a weakened heart by increasing the blood pressure to overcome vessel resistance and generate sufficient blood flow. Most VADs are designed as turbomachinery, with two primary forms: axial and radial types [3,4]. In both types, the impeller rotates with thousands of revolutions per minute, generating the needed pressures. However, the high rotational speeds lead to high-velocity gradients and, therefore, to supraphysiological stresses $\tau_{ij}=\mu \left(\left(\partial u_{i}\right)⁄\left(\partial x_{j}\right)+\left(\partial u_{j}\right)⁄\left(\partial x_{i}\right)\right)$. These stresses can damage the form and function of the blood components as they pass through the VAD [5]. Figure 1 displays simulation results of VAD flows, revealing that the highest stresses occur in the narrow gaps between the rotating impeller and the stationary housing [6]. As depicted in this figure, narrow gaps with the highest stresses can be identified in various VAD regions, such as bearings [7], tips of axial impellers [8], and side chambers of radial impellers [9]. These gaps typically measure only tens to hundreds of micrometers in height [5,9].

The compelling presence of these narrow gaps in VADs, coupled with the supraphysiological stresses within them, underscores the importance of flow optimization in these regions to improve the devices’ hemocompatibility. However, limited research has been conducted thus far to understand the real blood flow within narrow VAD gaps [5,7].

Nevertheless, comprehending blood flows in narrow gaps is crucial, as indicated by the existing literature for vascular research. Studies on flow dynamics in small vessels have revealed that red blood cells (RBCs) migrate and segregate from plasma in flows with geometric dimensions comparable to VAD gaps [10,11,12]. This migration leads to the formation of a low-viscosity near-wall layer, known as the cell-free layer (CFL), typically ranging from 300 to 7 ms [13]. During these processes, RBCs exhibit a localized distribution, clustering near the channel mid-plane while remaining absent in the CFL. This distribution significantly impacts flow dynamics, resulting in reduced shear stresses and pressure losses compared to fluid without particles/cells [14]. This influence of particle migration on blood flow in narrow gaps was first described by Fåhræus and Lindqvist in 1931 for vascular flows.

**Figure 2:**
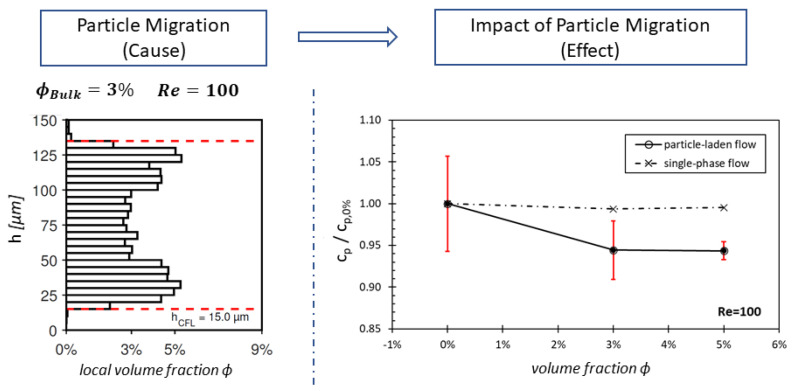
Results of the particle migration in gap flows at high Re from Knüppel et al. [15]. **Left:** Distribution of the local volume fraction $\phi_{loc}$ for a blood analog fluid (BAF) with a bulk volume fraction of ϕ=3%. **Right:** Normalized pressure loss coefficients $c_{p}$ across the gap flow at different particle volume fractions. The decrease in the black line compared to the dashed line with increasing volume fractions ϕ indicates smaller pressure losses due to particle migration compared to a single-phase flow. The percentage deviation between the particle-laden and single-phase flow is around 5% at ϕ=3%,5%, which also results in a 5% reduction in the apparent viscosity in the particle-laden flow.

To improve the hemocompatibility of future VADs, it is of great importance to develop a deeper understanding of the real blood flow conditions in the VAD gap. Cell migration in these gaps has not yet been researched deeply, but it is hypothesized that migration effects occur here [5,7,9]. For initial fundamental studies on CFL formation in VAD gaps, Knüppel et al. replicated the pressure-driven component of the gap flow in an experiment with geometric parameters and flow conditions similar to those in the VAD gaps [15], see Figure 2. They utilized optically accessible blood analog fluids (BAFs) with volume fractions up to ϕ=5% to investigate stresses, pressure losses, and viscosity reductions in the gap flow. The particles, which were used to create a particle-laden blood analog fluid, had different sizes. The first particles we used consisted of polystyrene (PS/Q-F L2459), with a diameter of 7.51 μm ± 0.09 μm. The second particles that we used were polymethylmethacrylate particles (PMMA-F-B1180) with a diameter of 7.52 μm ± 0.12 μm [16]. The size of these particles was chosen because red blood cells have a similar size in a range of 6 to 8 μm [17].

In contrast with Fåhræus and Lindqvist, this investigation was conducted at much higher Reynolds numbers $Re=(\rho \cdot c\cdot L/\mu_{\mathrm{rheo},\phi \%})∼100$, representing a flow regime similar to VAD gaps. In this Reynolds number definition, *c* represents bulk velocity, ρ and $\mu_{\mathrm{rheo},\phi \%}$ denote density and viscosity (measured in a rheometer), and *L* is the characteristic length. Similarly to Fåhræus and Lindqvist, the study of Knüppel et al. [15] revealed a significant reduction in stresses and pressure losses across the gap at high Re. Based on this experimental basic research, it was anticipated that a particle migration will also occur within real VAD gap flows, significantly influencing the flow dynamics in the gap.

However, as described above, the effect of particle migration is barely considered in VAD flow studies. For example, a single-phase fluid is often assumed in numerical VAD flow studies, which arises from the current limitations in computing technology, as direct consideration of the multiphase nature of blood in a VAD flow is not possible today [18]. This limitation stems from the disparity in scale between blood components and flow structures, necessitating significantly larger computational grids than those typically used in the current literature. Consequently, simulation methods like the boundary element method, immersed boundary method, lattice Boltzmann method, or lattice-free particle method, which require direct calculation of RBC behavior, become unfeasible due to resource constraints [19].

A solution to this problem has been proposed, e.g., by Stergiou et al. [10] for blood flows at Re≤6. Instead of attempting to directly compute individual RBCs, the authors suggested modeling the local volume fraction distribution resulting from the particle migration. Linking this volume fraction distribution to a viscosity model enables the determination of the local viscosity distribution in a gap flow with particle migration. This local viscosity adjustment enables the easy incorporation of the effects of the particle migration into the flow field in a VAD simulation.

Yet, no viscosity model specifically tailored for VAD gap flows at high Reynolds numbers exists, since the available models are derived for significantly smaller Reynolds numbers (Re∼1) [20,21]. Hence, we aim to address this research gap by deriving a viscosity model for high Re based on our previously assessed experimental results. This model will enable the computation of the local particle distribution in the gap flow, represented by a spatially distributed viscosity, and will allow us to assess its impact on pressure loss and stresses in high Reynolds number gap flows. The proposed viscosity model will be valid for Reynolds numbers ranging from Re=50 to 150, which typically occur in the narrow gaps of VADs, a gap height of H=150μm, and particle volume fractions up to ϕ=5%.

It will be shown that this viscosity model is valid for both blood analog fluids and porcine blood up to a particle volume fraction of ϕ=5%. Regarding this, Calejo et al. [22] and Pinho et al. [23] showed in an experimental study for these particle volume fractions that the height of the CFLs formed for blood analog fluids and blood are very close to each other.

## 2. Materials and Methods

### 2.1. Starting Point and Model Development

The viscosity model is based on the results of a collaborative project between the University of Rostock and TU Darmstadt. CFL heights as well as local particle distributions were measured by astigmatism particle tracking velocimetry (APTV), performed at TU Darmstadt [24,25]. Additionally, we provided indirect evidence for the CFL formation through the wall shear stress and pressure measurements at the University of Rostock.

Starting Point

This section details the development of our viscosity model. The foundation of the model lies in the experimental analysis of particle distribution within a particle-laden gap flow operating at high Reynolds numbers. For a comprehensive understanding of the experimental setup, readers are directed to our earlier publication in Knüppel et al. [15].

The particle distribution is influenced by the cross-sectional shape of the channel. However, we focus on the leakage flow within the gap in a ventricular assist device (VAD), where the height is much smaller than the width. We approximate this leakage flow as an infinitely wide plane channel, using a straight plane channel with a width-to-height ratio exceeding 7:1. Due to the significant width-to-height ratio of the channel, the effect of the channel’s cross-sectional shape on the particle distribution can be ignored. For this reason, we only focus on the distribution of particles in height.

We utilized APTV to determine the particle distribution $\phi_{loc}\left(h\right)$ of a blood analog fluid (BAF) within a microchannel with a height of H=150μm, covering Reynolds numbers ranging from 50 to 150. Furthermore, the rheological properties were integral to our study. Hence, we measured both the dynamic viscosity of the analyzed fluid, denoted as $\mu_{\mathrm{rheo},\phi \%}$ at a specific bulk volume fraction ϕ, and the viscosity of the carrier fluid, represented by $\mu_{\mathrm{carrier}}\;\left(=\mu_{\mathrm{rheo},0\%}\right)$. In the case of blood, this refers to plasma without the presence of cells. It is important to determine $\mu_{\mathrm{carrier}}$, since it represents the viscosity in the CFL.

(1) $$\mu_{loc}\left(h\right)=\mu_{carrier}{\left(1-1.35\cdot \phi_{loc}\left(h\right)\right)}^{-2.5}$$

**Figure 3:**
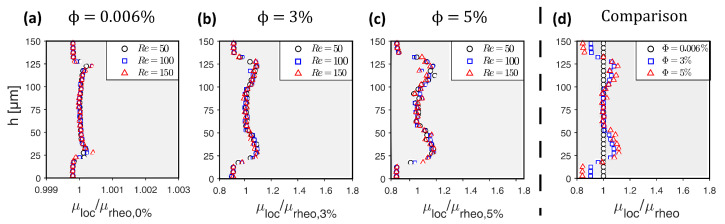
Local viscosity distributions $\mu_{loc}\left(h\right)$ for different bulk particle volume fractions ϕ along channel height *h* with measured CFL at h≤20μm and h≥130μm. The viscosity is normalized with the dynamic viscosity $\mu_{\mathrm{rheo},\phi \%}$ from the rheometer measurements.

To establish a connection between the particle distribution and rheological measurements, we derived a local, height-dependent viscosity distribution $\mu_{\mathrm{loc}}\left(h\right)$. This was achieved through the application of the Einstein–Roscoe Equation (1) [26]. The resulting viscosity distributions are illustrated for various particle volume fractions ϕ in Figure 3. This figure also shows that the height of the CFL formed independently of the Reynolds number.

Development of the Viscosity Model

The code for our viscosity model is open-access, written in MATLAB 2020b, and can be found in a repository [27]. We implemented our viscosity model as an additional equation in the commercial solver ANSYS CFX 2022R2 (ANSYS Inc., Pittsburgh, PA, USA), incorporating it as an expression to define heterogeneous viscosity properties. The creation of this expression requires several input values, detailed in Table 1.

**Figure 4:**
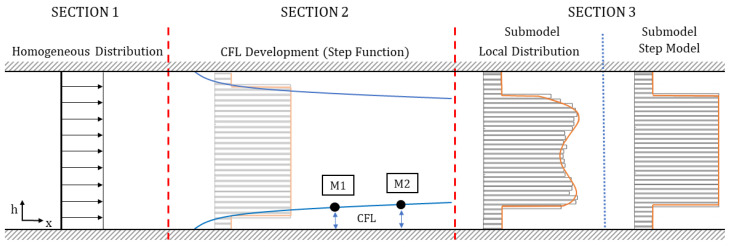
Schematic visualization of the different sections for the CFL development in the viscosity model.

The viscosity model comprises distinct sections, illustrating the progression of a particle-laden flow in a gap. These sections encompass the evolution from a homogeneous particle distribution (Section 1) to an intermediate stage where particle migration occurs and the CFL develops (Section 2), ultimately culminating in a final state where particles reach equilibrium positions and a steady-state CFL is formed (Section 3). These sections are visualized in Figure 4.

Section 1—Homogeneous Distribution: The viscosity model is designed in such a way that the viscosity does not change only with channel height *h*, but also in the flow direction (*x*-axis), representing a particle-laden flow, which starts flowing from a homogeneous distribution. In Section 1, a homogeneous particle distribution is defined based on $\mu_{\mathrm{rheo},\phi \%}$. It is assumed that this distribution will exist only over a short distance in flow direction, e.g., just for one micron. The definition of this length for the homogeneous distribution is empirical and is necessary for generating the CFL development discussed in Section 2.
  (2) $$\mu_{loc}\left(h,x\right)=\mu_{rheo,\phi \%}=const.,\;for\;x<1\;\mu m$$
  Section 2—CFL Development: The literature has shown that particle-laden flows exhibit a development length for particle migration to reach an equilibrium position and establish a steady-state CFL height [28].

As noted by Coupier et al. [29], a tilted parabolic function can be employed to describe the CFL formation during this development. In our study, the CFL formation was monitored by two APTV measuring planes in the microchannels. Based on the two measuring planes (M1 and M2 in Figure 4), Equation (3) is formulated to define the CFL development in flow direction. In this equation, constant *a* denotes the widening of the parabolic function, which is calibrated by M1 and M2. The CFL formation is considered complete when the change in CFL height is less than 0.1% in *x*-direction.

**Figure 5:**
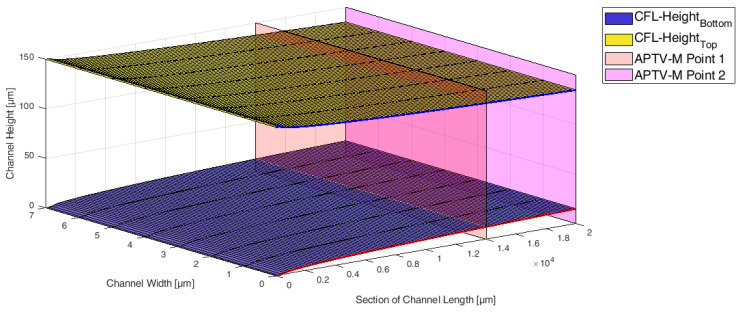
Development length of the CFL at a Reynolds number of Re=100 in a microchannel. The tilted parabolic equation from Equation (3) is plotted. Additionally, the two APTV measurement planes for calibrating the parabolic function are included.

Figure 5 illustrates the CFL formation and the two optical measurement planes for Reynolds number 100 and a particle volume fraction of 3%. It is assumed that the local viscosity in the CFL equals the viscosity of the carrier fluid $\mu_{\mathrm{carrier}}$.

(3) $$H_{CFL}\left(x\right)=\sqrt{a\cdot x-1\;\mu m},\;for\;x>1\;\mu m$$

After defining the CFL height and the viscosity within it, it becomes crucial to account for the viscosity distribution for the remaining channel height, where particles are present and contribute to increased viscosity. This is achieved by assuming a still uniform distribution of particles outside of the CFL at a given position *x*, which is a simplified modeling approach. In this context, the entire viscosity distribution $\mu_{\mathrm{loc}}\left(h,x\right)$ of the gap can be described by a step function (Equation (4)) between the low viscosity $\mu_{\mathrm{carrier}}$ within the CFL and the high viscosity $\mu_{STEP\_particles}$ outside the CFL.

(4) $$\mu_{loc}\left(h,x\right)=\left\{\begin{array}{cc} \mu_{\mathrm{carrier}}, & if\;\;h<H_{CFL}\left(x\right)\;@\;\mathrm{bottom}\;\mathrm{of}\;\mathrm{channel}\\ \mu_{\mathrm{STEP}\_\mathrm{particles}}\left(x\right), & if\;\;h>H_{CFL}\left(x\right)\;@\;\mathrm{bottom}\;\mathrm{of}\;\mathrm{channel}\\ \mu_{\mathrm{carrier}}, & if\;\;h>H-H_{CFL}\left(x\right)\;@\;\mathrm{top}\;\mathrm{of}\;\mathrm{channel} \end{array}\right.$$

The final step involves defining $\mu_{\text{STEP\_particles}}\left(x\right)$. This can be achieved using Equation (5):

(5) $$\mu_{\text{STEP\_particles}}\left(x\right)=\frac{H\cdot \mu_{\mathrm{rheo},\phi \%}-H_{CFL}\left(x\right)\cdot \mu_{\mathrm{carrier}}}{H-H_{CFL}\left(x\right)}$$

**Figure 6:**
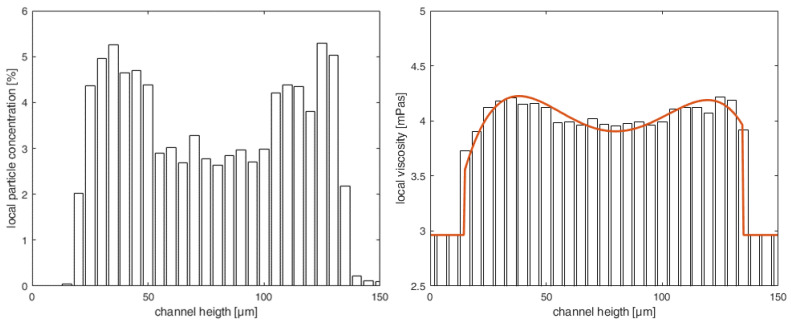
**Left:** Local particle distribution of the bulk volume fraction ϕ=3% for a blood analog fluid at Re=100. **Right:** Local viscosity distribution $\mu_{loc}\left(h\right)$ with step function of the CFL and subsequent polynomial function.

Section 3—Heterogeneous distribution with steady-state CFL: Once the equilibrium position of the particles is attained and a steady-state CFL is established, the optically measured data from particle distributions (see Figure 3 and Figure 6) are utilized to define the heterogeneous viscosity distributions for the simulations.

In Section 3, two submodels were developed to represent the viscosity distribution for a particle-laden flow with steady-state CFL. The first submodel is based on the measured *Local Distribution* of $\mu_{loc}\left(h\right)$, as depicted in Figure 6. Here, a fourth-degree polynomial function (illustrated by the orange line in Figure 6) is utilized to characterize the viscosity distribution $\mu_{loc}\left(h\right)$.

Given that certain fluids with suspended particles (e.g., PEG–water mixtures with polystyrene particles) may deviate from the predicted viscosity distribution by the Einstein–Roscoe Equation (1), an adjustment factor was incorporated into our viscosity model). This adjustment ensures alignment between $\mu_{\mathrm{rheo},\phi \%}$ and the height-averaged value $\frac{1}{H}\int_{0}^{H}\left(\mu_{\mathrm{loc}}\right)dh$, thereby maintaining consistency between these two values.

Also, a second submodel termed the *Step Model*, is necessary because, in the case of blood, it may not be feasible to measure the entire particle distribution due to optical limitations. For model development, we hypothesized that primarily the formation of the CFL leads to changes in flow dynamics in the particle-laden flow. Hence, minor variations in the viscosity distribution outside the CFL do not play a role in accurately determining stress and pressure loss reduction in the microchannel. Therefore, the viscosity distribution outside the CFL was assumed as a constant value, as shown in the right subplot in Figure 4. While implementing this approach, we ensure once more that the integration of the local viscosity $\mu_{\mathrm{loc}}$ over the channel height, represented by $\frac{1}{H}\int_{0}^{H}\left(\mu_{\mathrm{loc}}\right)dh$, remains consistent with the rheologically measured dynamic viscosity $\mu_{\mathrm{rheo},\phi \%}$.

### 2.2. Numerical Setup for the Simulations with the Novel Viscosity Model

**Figure 7:**
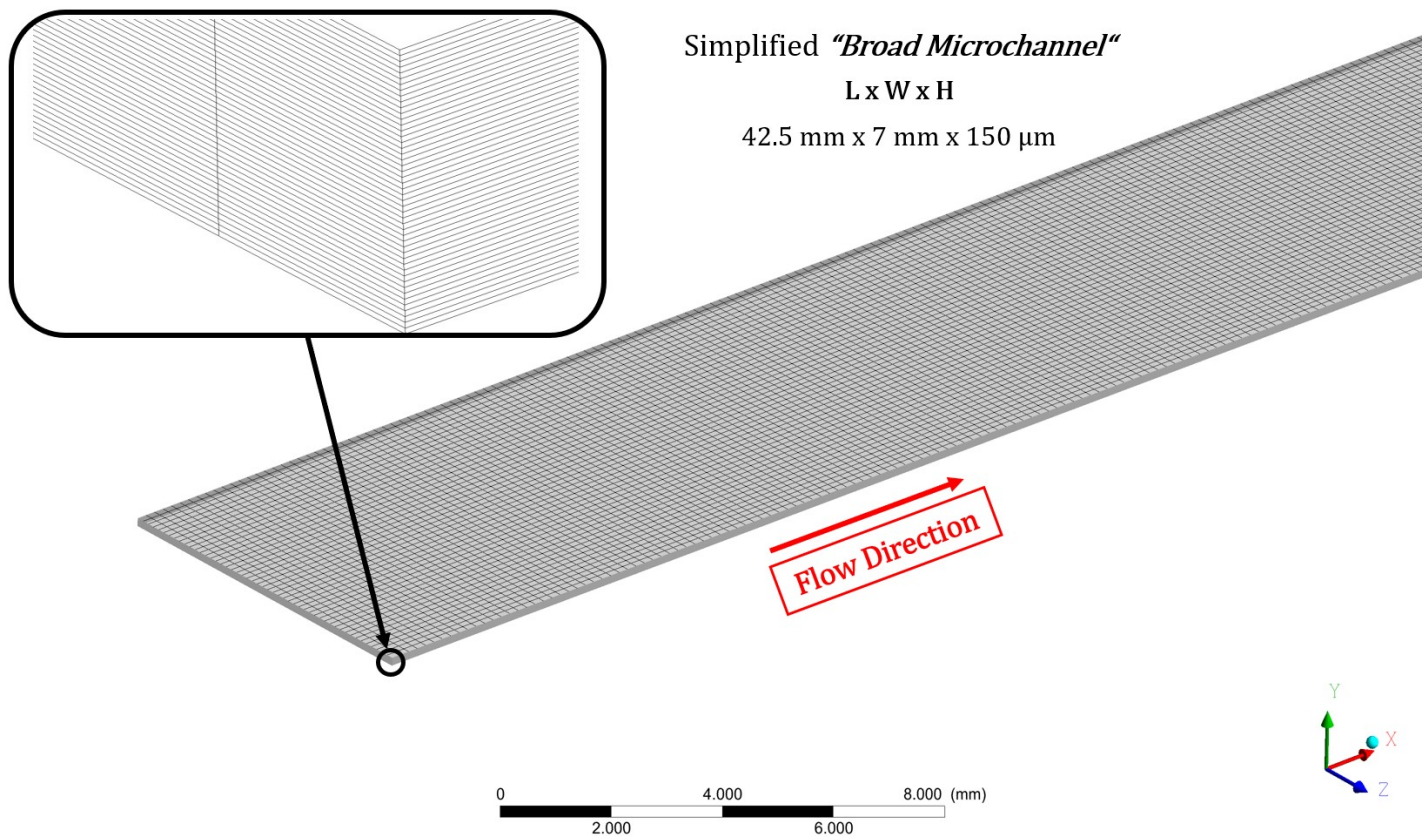
Computational domain of the microchannel, which is used for the simulations.

We conducted simulations of the particle-laden flow through a microchannel, representing a narrow gap flow in laboratory conditions, using our viscosity model. We performed direct numerical simulations (DNSs) across Reynolds numbers ranging from 50 to 150. The computational domain of the microchannel closely resembles those used in the experiments conducted by Knüppel et al. [15], and it is illustrated in Figure 7. The computational grid comprises 1.25 million nodes and features an aspect ratio of 65, grid angles of 45°, and a volume change of 1.

### 2.3. Testing and Validation of the Viscosity Model

To test and validate the viscosity model, the simulations were compared with data from two different measurement campaigns. The first campaign involved BAF, and these measurements are comprehensively reported in Knüppel et al. [15]. In this campaign, wall shear stresses (WSSs) and pressure losses in a particle-laden flow through a microchannel at different Reynolds numbers were conducted for ϕ≤5%.

A second measurement campaign was realized with porcine blood with a particle volume fraction (hematocrit) of ϕ=5% to prove that the viscosity model is also valid for blood. For this purpose, porcine blood was obtained from the *Research Institute for Farm Animal Biology (FBN)* and then adjusted to the appropriate volume fraction at the *Institute of Clinical Chemistry and Laboratory Medicine, Rostock University Medical Center*. Then, the rheological viscosity measurements for $\mu_{\mathrm{rheo},\phi \%}$ of the blood were conducted using an *AntonPaar MCR302e* (*Anton Paar Group AG, Graz, Austria*). Despite blood being generally considered a non-Newtonian fluid, the shear rates in our microchannels at the investigated Reynolds numbers are high enough (shear rates of ≈(400–60,000) 1/*s* for Re=150 and blood at ϕ=5%) to treat blood as Newtonian [30]. Therefore, in our simulations, only the asymptotic blood viscosity value at high shear rates was taken as $\mu_{\mathrm{rheo},\phi \%}$.

For the pressure loss experiments, measurement uncertainties were included. These uncertainties were obtained using the following equation [31]:

(6) $$u_{x}=x_{s}+\tau \cdot s_{x}$$

$x_{s}$ is the estimated maximum amount of the systematic error and was set to 2000Pa. Furthermore, $\tau \cdot s_{x}$ represents the confidence limit and was calculated with a confidential level of 95%. τ varies with the number of measurements performed. $s_{x}$ describes the standard deviation of the mean value [32].

## 3. Results and Discussion

We will conduct a comparative analysis between the simulation results obtained using the viscosity model and the measured data for wall shear stresses (WSSs) and pressure losses. This comparison will involve analyzing the stress field within the particle-laden flow. To evaluate the influence of CFL formation on the overall stress distribution, we will further compare the computed stress field with that of a simulation assuming single-phase flow with spatially constant viscosity.

### Testing and Validating the Viscosity Model against Experimental Data

In Table 2, the experimentally assessed WSS of BAF and blood are compared against the simulation results from the submodels *Local Distribution* and *Step Model*. As explained in the methods, only the *Step Model* was considered in blood. Additionally, the table presents WSS results from simulations considering a single-phase flow. Each experimental wall shear was measured between five and seven times, and the mean value was calculated from these measurements.

Considering all cases, it becomes evident that simulations with the viscosity model generally provide closer agreement with WSS experiments compared to simulations assuming a single-phase flow. Particularly for the high WSS values in the BAF, simulations assuming a single-phase flow result in markedly overestimated WSS. This discrepancy is attributed to the unaccounted effect of particle migration in the “single-phase CFD”. Particle migration leads to the formation of a CFL, wherein the viscosity is reduced to $\left(\mu_{\mathrm{carrier}}\right)$, resulting in smaller stresses [15], as shown in Equation (7). In contrast, CFD simulations incorporating the viscosity model consider the CFL formation, thus accurately accounting for WSS.

(7) $$\tau_{ij}{|}_{CFL}=\mu_{\mathrm{carrier}}\cdot \left(\frac{\partial u_{i}}{\partial x_{j}}+\frac{\partial u_{j}}{\partial x_{i}}\right)$$

**Figure 8:**
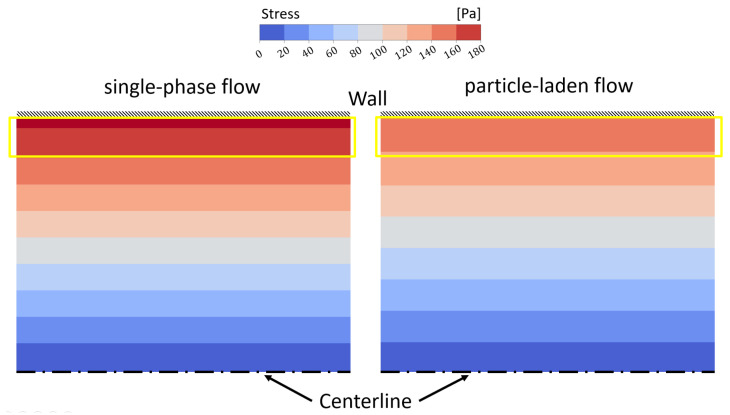
**Left:** Stress distribution in a single-phase flow assuming a homogeneous viscosity distribution. **Right:** Stress distribution in a particle-laden flow with an inhomogeneous viscosity distribution due to the particle migration. Computed for the BAF at ϕ=3% and Re=100. The CFL region (only present in the right subfigure) is sketched as a yellow frame in this figure.

The impact of particle migration and CFL formation on the entire stress field in a particle-laden flow is illustrated in Figure 8. Compared to the single-phase flow, the stresses are not only reduced directly at the walls but also throughout the entire channel.

The same trend as described above for the BAF can be observed for the simulations with blood at ϕ=5%. Nonetheless, both simulations with single-phase flow and those with the viscosity model are relatively close to the experimental results, as shown in Table 2, while the former tends to overpredict and the latter tends to slightly underpredict WSS. The similarity in both simulated WSS can be explained by considering the viscosity difference of the “whole” fluid and those just within the CFL $\left(\mu_{rheo,\phi \%}-\mu_{\mathrm{carrier}}\right)$. This difference is relatively small at hematocrits of 5%, with the following values: plasma: μ=1.38 mPa·s; blood ϕ=5%: μ=1.45 mPa·s, resulting in a difference of 1.45−1.38=0.07 mPa·s for blood. Hence, the effect of stress reduction due to viscosity decrease in the CFL is not as pronounced as with the BAF at ϕ=5%, where the viscosity difference is markedly higher, at 3.1 mPa·s (carrier fluid:μ=2.85 mPa·s; BAF ϕ=5%: μ=5.95 mPa·s).

Based on this observation, we postulate that the impact on stress reduction becomes more pronounced in blood flows characterized by higher particle volume fractions. This can be illustrated by considering a hypothetical scenario based on our rheological measurements: At higher particle volume fractions, e.g., ϕ=45%, the difference between plasma viscosity and blood viscosity will increase (plasma: μ=1.38 mPa·s, blood ϕ=45%: μ=3.63 mPa·s). Consequently, we can infer that as the volume fraction increases, the CFL’s effect on stress reduction will become more pronounced, underscoring the necessity for a proper modeling approach at high hematocrits such as our viscosity model.

**Figure 9:**
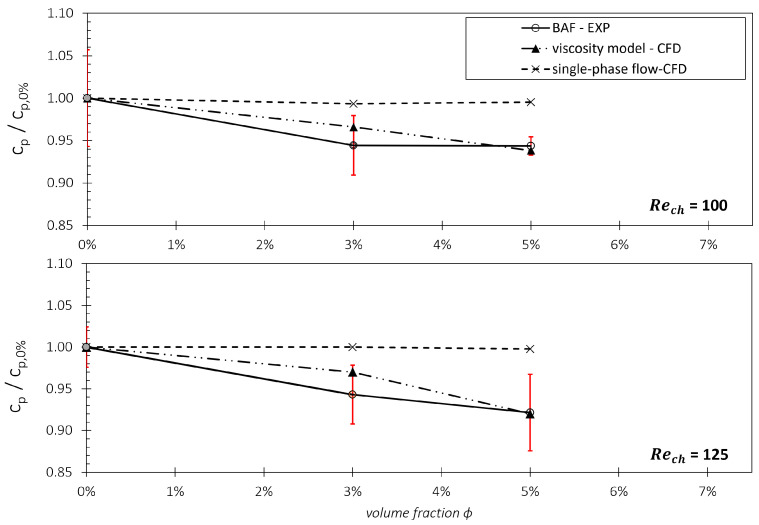
Normalized pressure loss coefficients $c_{p}$ in the microchannels for the blood analog fluid (BAF) at different volume fractions and Reynolds numbers. The red lines indicate the measurement uncertainties. Comparison of the simulation and the experiment.

**Figure 10:**
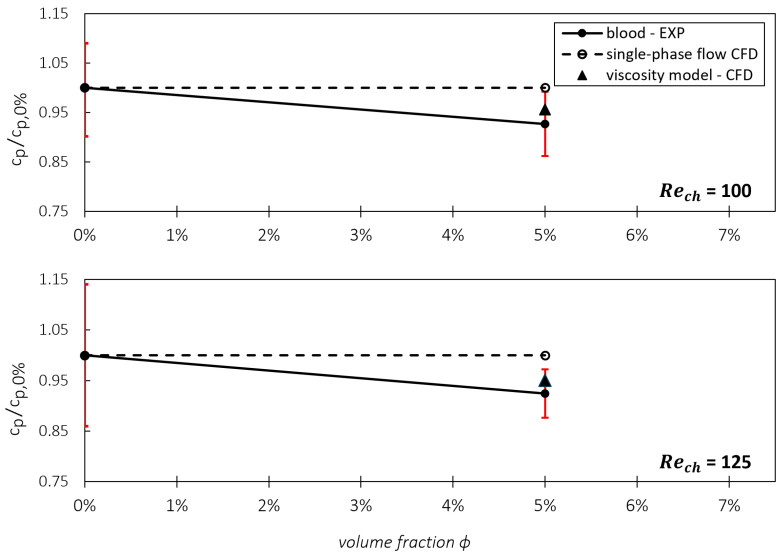
Normalized pressure loss coefficients $c_{p}$ in the microchannel with porcine blood at a volume fraction of 5% for two different Reynolds numbers. The red lines indicate the measurement uncertainties.

In addition to examining the wall shear stresses directly, we explored whether the viscosity model can accurately reproduce the overall impact of stress reduction resulting from CFL formation on pressure losses in the microchannel. Therefore, we compared the simulated pressure loss through the channel with experimental data from Knüppel et al. [15], as depicted in Figure 9 and Figure 10. In these figures, the pressure loss is represented as a pressure loss coefficient $c_{p}$, which is further normalized by $c_{p,0\%}$, denoting the $c_{p}$-value when only the carrier fluid flowing through the channel. By this normalization, we can compare pressure loss reductions in the particle-laden flow more descriptively for different ϕ and Re. As depicted in Figure 9, the CFD utilizing the single-phase assumption results in a constant value of $c_{p}/c_{p,0\%}=1$ (illustrated by dotted lines).

While this constant value aligns with the expected progression of a dimensionless number in single-phase flows for a specific Reynolds number, it fails to capture the flow dynamics observed in multiphase flow conditions, as evidenced by experimentally assessed pressure losses. The experimentally observed decrease in pressure losses with increasing particle volume fraction ϕ can be attributed to the increasing prominence of the low viscosity in the CFL region. This prominence leads to reduced stresses and, consequently, reduced dissipation losses compared to those losses in a single-phase flow. Importantly, our viscosity model successfully simulates this trend in pressure loss reduction for the BAF with increasing ϕ, with the simulated value falling within the uncertainty interval of the experiments.

In Figure 10, the normalized $c_{p}$-values of the blood experiments are compared with those of the CFD with viscosity model and with single-phase flow assumption. Here, plasma from the porcine blood was used as the particle-free fluid for the measurements. Again, a close match between the results of the viscosity model and the experimentally assessed results can be seen and the trend observed in the blood experiments mirrors that of the blood analog fluids. Interestingly, the results in Figure 9 and Figure 10 show a Reynolds-number-independent trend for all shown experiments in blood and BAF. The reason for this Reynolds number independence was discussed in a previous publication by Knüppel et al. [15].

Considering the measurement results for blood, the averaged $c_{p}/c_{p,0\%}$-value for both Reynolds numbers is approximately 0.93. If the $c_{p}$-value is now computed using the viscosity model, the results are $c_{p}/c_{p,0\%}=0.956$ for Re=100 and a $c_{p}/c_{p,0\%}=0.951$ for Re=125. These values lie within the uncertainty interval of the blood measurements and are close to the mean experimental values (block dots). Once again, the CFD data with the viscosity model are closer to the measured $c_{p}$-values of the experiments compared to the CFD results obtained from the single-phase assumption. The results for the CFD with single-phase flow assumption lead to a relative deviation of circa 5% to the measurement results and lie outside of the uncertainty interval.

The analysis of Figure 9 and Figure 10 demonstrates that the viscosity model performs consistently across different Reynolds numbers, with no statistically significant differences to each other. This consistency, combined with the alignment between experimental and simulation data, underscores the robustness of the model and its potential for widespread application in technical settings involving narrow gaps and high Reynolds numbers, such as in ventricular assist devices (VADs). The observed trends indicate that the viscosity model accurately captures the effects of particle concentration on the normalized pressure coefficient ($c_{p}/c_{p,0\%}$), and the minimal variation across Reynolds numbers suggests that the model may be generalizable to different flow conditions without substantial recalibration.

## 4. Limitations

Unless the viscosity model demonstrates a significant improvement for simulating particle-laden flows compared to the single-phase flow assumption, it has limitations that should be addressed. In this respect, the model is currently only valid for flows in narrow gaps of approximately 150μm and volume fractions ≤5%. As mentioned in the introduction, Calejo [22] and Pinho [23] have described similar heights in CFL formation for ϕ = 5% (blood and blood analog fluid). Since it is also known that at higher blood particle concentrations the viscosity does not follow the Einstein–Roscoe equation, this equation has to be adapted for higher hematocrits. Nevertheless, the current script should be valid for higher particle concentrations of BAF. This has not yet been investigated further.

While the investigated small volume fractions are essential for basic research to understand blood flow at different ϕ, it does not reflect the physiological hematocrit. The next crucial step in our research would be to expand the viscosity model to higher volume fractions. Therefore, experiments with BAF and blood are planned to increase up to 40%. This will allow us to test our hypothesis that the deviations of WSS predicted by CFD assuming single-phase flow will increase compared to experimental results with increasing ϕ.

Our generic viscosity model, designed for technical applications with narrow gaps and high Reynolds number flows, is intended for future application in all types of ventricular assist devices (VADs). Currently, it is being developed and validated under laboratory conditions in microchannels that simulate VAD flow environments. The preliminary results are promising, demonstrating the model’s ability to simulate particle migration effects on wall shear stress and pressure loss. Future work will focus on verifying the model’s accuracy in real VAD flow conditions.

## 5. Conclusions

This work aimed to develop a viscosity model that uses the local particle distribution to determine a corresponding local viscosity distribution, thereby enabling the simulation of the multiphase nature of particle-laden gap flows. The developed viscosity model constitutes an initial endeavor toward numerically characterizing particle migration in narrow gaps under high Reynolds numbers Re = 50–150, mirroring flow conditions encountered in ventricular assist devices.

The results demonstrate that simulations incorporating the viscosity model exhibit strong agreement with measurements conducted in a blood analog fluid (BAF) and blood up to a particle volume fraction of 5%. Conversely, employing a single-phase flow assumption in the simulation led to greater deviations in both stresses and pressure losses for a particle-laden flow.

Modeling the migration effects at high-volume fractions using an extended version of our viscosity model would be a next crucial step. Importantly, our model can then be readily applied to flow simulations of VADs and accurately predict shear stresses in the gaps of these devices. Hence, more reliable assessments regarding the hemocompatibility of a VAD can be made in future CFD studies.

## Figures and Tables

**Table 1:** Input variables necessary for defining the viscosity model.

Input Variables	Units
Channel height (*H*)	μm
CFL height ($H_{CFL}$)	μm
Viscosity of the particle-laden fluid ($\mu_{\mathrm{rheo},\phi \%}$)	mPas
Viscosity of the carrier fluid ($\mu_{carrier}$)	mPas
Local particle distribution ($\phi_{loc}\left(h,x\right)$)	%

**Table 2:** Comparison of the wall shear stresses between experiments (EXP) and the simulations (CFD) with relative deviations to the experiments in brackets.

Fluid	ϕ	Re	Wall Shear Stresses (WSS) $\tau_{w}$			
			Ø *EXP*	*CFD—Single*	*CFD with Viscosity Model +*	
				*Phase*	*Local Distribution*	*Step Model*
**Blood Analog**	ϕ=3%	100	167 Pa	189 Pa (+12%)	171 Pa (+2%)	171 Pa (+2%)
		150	257 Pa	283 Pa (+9%)	257 Pa (±0%)	257 Pa (±0%)
**Fluid (BAF)**	ϕ=5%	50	166 Pa	225 Pa (+36%)	169 Pa (+2%)	169 Pa (+2%)
**Blood**	ϕ=5%	100	42 Pa	45 Pa (+7%)	−	41 Pa (−2%)
		125	55 Pa	56 Pa (+2%)	−	52 Pa (−6%)
		150	63 Pa	67 Pa (+6%)	−	61 Pa (−3%)

## Data Availability

The original contributions presented in the study are included in the article, further inquiries can be directed to the corresponding author.

## Nomenclature and Abbreviations

The following nomenclature are used in this manuscript:

*c*	Bulk Velocity	[m/s]
$c_{p}$	Pressure Coefficient	[-]
$c_{p}/c_{p,0\%}$	Normalized Pressure Coefficient	[-]
$D_{h}$	Hydraulic Diameter	[m]
H,h	Channel Height	[m]
$H_{CFL}$	Height Cell-Free Layer	[m]
*L*	Characteristic Length	[m]
Re	Channel Reynolds Number	[-]
$u_{i},u_{j}$	Velocities	[m/s]
*w*	Channel Width	[m]
$x_{i},x_{j}$	Spatial Directions	[m]
$\mu_{app}$	Apparent Viscosity	[mPas]
$\mu_{carrier}$	Carrier Viscosity	[mPas]
$\mu_{STEP_{particles}}$	Viscosity of the Region with Particles	[mPas]
$\mu_{loc}$	Local Viscosity	[mPas]
$\mu_{rheo,\phi \%}$	Bulk Viscosity (from the Rheometer)	[mPas]
ρ	Density	[kg/m^3^]
$\tau_{ij}$	Shear Stress	[Pa]
$\tau_{w}$	Wall Shear Stress	[Pa]
ϕ	Particle Volume Fraction	[%]
$\phi_{loc}$	Local Particle Distribution	[%]
APTV	Astigmatism Particle Tracking Velocimetry	
BAF	Blood Analog Fluid	
CFD	Computational Fluid Dynamics	
CFL	Cell-Free Layer	
DNS	Direct Numerical Simulation	
EXP	Experiment	
PEG-water	Polyethylene Glycol–Water Mixture	
RBC	Red Blood Cell	
VAD	Ventricular Assist Device	
WSS	Wall Shear Stress	
